# Supplementary material for: The role of lactate dehydrogenase in hospitalized patients, comparing those with pulmonary versus non-pulmonary infections: A nationwide study
Source: PLoS One. 2023 Mar 29;18(3):e0283380. doi: 10.1371/journal.pone.0283380 (PMC10058092; doi:10.1371/journal.pone.0283380)
Supplement: S1 File — (DOCX) [file pone.0283380.s001.docx]

| **Table 1:** Demographic characteristics and hospitalization details | | | |
| --- | --- | --- | --- |
|  | **Infection from pulmonary source**  n=44,491 | **Infection from other sources**  n=50,486 | **P-value** |
| Age, mean ± SD | 70.7 ± 17.3 | 68 ± 19.4 | <0.001 |
| Male sex, No. (%) | 23226 (52.2) | 23568 (46.7) | <0.001 |
| Deceased, No. (%) | 26651 (59.9) | 26031 (51.6) | <0.001 |
| Age, mean ± SD | 81.1 ± 11.9 | 81.8 ± 11.4 | <0.001 |
| In hosp, No. (%) | 1846 (6.9) | 890 (3.4) | <0.001 |
| One week disch, No. (%) | 478 (1.8) | 427 (1.6) | 0.18 |
| One month disch, No. (%) | 1782 (6.7) | 1779 (6.8) | 0.5 |
| ICU transfer, No. (%) | 934 (2.1) | 547 (1.1) | <0.001 |
| Hospitalization length of stay, median (IQR) | 5 (3-7) | 5 (3-7) | <0.001 |
|  |  |  |  |
|  | | | |

| **Table 2: Target organ damage** | | | |
| --- | --- | --- | --- |
|  | **Infection from pulmonary source**  **n=44,491** | **Infection from other sources**  **n=50,486** | **P-value** |
| **Respiratory failure, No. (%)** | **2341 (5.3)** | **522 (1)** | **<0.001** |
| **Vascular, No. (%)** | **303 (0.7)** | **313 (0.6)** | **0.24** |
| **Renal, No. (%)** | **3074 (6.9)** | **4194 (8.3)** | **<0.001** |
| **Liver, No. (%)** | **59 (0.1)** | **336 (0.7)** | **<0.001** |
| **Hematologic, No. (%)** | **617 (1.4)** | **631 (1.2)** | **0.06** |
| **Metabolic, No. (%)** | **358 (0.8)** | **71 (0.1)** | **<0.001** |
| **CNS, No. (%)** | **24 (0.1)** | **79 (0.2)** | **<0.001** |
| **SIRS, No. (%)** | **322 (0.7)** | **252 (0.5)** | **<0.001** |
| **Any target organ damage, No. (%)** | **6230 (14)** | **5810 (11.5)** | **<0.001** |
|  | | | |

| **Table 3:** Background medical conditions | | | |
| --- | --- | --- | --- |
|  | **Infection from pulmonary source**  n=44,491 | **Infection from other sources**  n=50,486 | **P-value** |
| DM | 16147 (36.3) | 18821 (37.3) | 0.002 |
| IHD | 16070 (36.1) | 15516 (30.7) | <0.001 |
| CVA | 2348 (5.3) | 2954 (5.9) | <0.001 |
| Hematologic malignancy | 2350 (5.3) | 1812 (3.6) | <0.001 |
| Solid malignancy | 24835 (55.8) | 27775 (55) | 0.01 |
| Liver cirrhosis | 534 (1.2) | 919 (1.8) | <0.001 |
| CHF | 10899 (24.5) | 8434 (16.7) | <0.001 |
| CKD/renal failure | 10151 (22.8) | 11435 (22.6) | 0.54 |
| COPD | 15304 (34.4) | 6200 (12.3) | <0.001 |
| HTN | 28915 (65) | 31893 (63.2) | <0.001 |
| Asthma | 8111 (18.2) | 4649 (9.2) | <0.001 |
| A. Fib/Flutter | 9596 (21.6) | 8114 (16.1) | <0.001 |
|  | | | |

| **Table 4:** Chronic medication use | | | |
| --- | --- | --- | --- |
|  | **Infection from pulmonary source**  n=44,491 | **Infection from other sources**  n=50,486 | **P-value** |
| BB | 15156 (34.1) | 16127 (31.9) | <0.001 |
| ACEi | 13103 (29.5) | 14917 (29.5) | 0.75 |
| ARB’s | 5246 (11.8) | 5161 (10.2) | <0.001 |
| Steroids | 7367 (16.6) | 4648 (9.2) | <0.001 |
| Statins | 16674 (37.5) | 17828 (35.3) | <0.001 |
| Altroxin | 2775 (6.2) | 2951 (5.8) | 0.01 |
| Purchased three months before hospitalization | | | |

| **Table 5:** Lab tests results during hospitalization (limiting values) | | | |
| --- | --- | --- | --- |
|  | **Infection from pulmonary source**  n=44,491 | **Infection from other sources**  n=50,486 | **P-value** |
| Hb (min), gr/dL, mean ± SD | 11.4 ± 1.9 | 11.2 ± 1.9 | <0.001 |
| Hb < 6, No. (%) | 78 (0.2) | 95 (0.2) | 0.67 |
| Hb < 7, No. (%) | 495 (1.1) | 569 (1.1) | 0.9 |
| Hb < 9, No. (%) | 4888 (11.1) | 5997 (12) | <0.001 |
| WBC (max), 10^3^/µL, median (IQR) | 11.9 (8.8-16.2) | 12.6 (9.2-16.8) | <0.001 |
| WBC (min), 10^3^/µL, median (IQR) | 8.1 (6.1-10.5) | 7.9 (6-10.3) | <0.001 |
| WBC > 15, No. (%) | 13476 (30.6) | 17065 (34) | <0.001 |
| WBC < 5, No. (%) | 6019 (13.9) | 6670 (13.4) | 0.06 |
| PLT (min), 10^3^/µL, mean ± SD | 219.2 ± 87.5 | 212.5 ± 83.2 | <0.001 |
| PLT < 50, No. (%) | 742 (1.7) | 622 (1.3) | <0.001 |
| INR (max), median (IQR) | 1.1 (1-1.3) | 1.1 (1-1.3) | <0.001 |
| Glucose (max), mg/dL, median (IQR) | 147 (120-195) | 140 (115-186) | <0.001 |
| Glucose (min), mg/dL, median (IQR) | 97 (84-117) | 94 (82-112.7) | <0.001 |
| Glucose > 200, No. (%) | 10175 (23.3) | 10186 (20.6) | <0.001 |
| Glucose < 50, No. (%) | 545 (1.3) | 719 (1.5) | 0.008 |
| Glucose < 70, No. (%) | 3105 (7.1) | 4463 (9) | <0.001 |
| Glucose coefficient of variance, median (IQR) | 0.2 (0.1-0.3) | 0.2 (0.1-0.3) | 0.008 |
| AST (max), U/L, median (IQR) | 26 (19-41) | 25 (18-41) | <0.001 |
| ALT (max), U/L, median (IQR) | 22 (15-38) | 21 (14-36) | <0.001 |
| Creatinine (max), mg/dL, median (IQR) | 1 (0.8-1.4) | 1 (0.8-1.4) | <0.001 |
| Maximal CRE/first CRE ratio, median (IQR) | 1 (1-1.1) | 1 (1-1) | <0.001 |
| Maximal CRE/first CRE > 2, No. (%) | 515 (1.2) | 451 (0.9) | <0.001 |
| D-Dimer (max), ng/mL, median (IQR) | 656 (5.6-1509.9) | 450.5 (1.7-1576) | 0.002 |
| Urea (max), mg/dL, median (IQR) | 47 (33-72) | 44 (30.5-67) | <0.001 |
| Albumin (min), gr/dL, median (IQR) | 3.3 (2.9-3.7) | 3.3 (2.9-3.6) | <0.001 |
| Albumin < 1.5, No. (%) | 43 (0.1) | 46 (0.1) | 0.86 |
| Albumin < 2, No. (%) | 720 (1.7) | 909 (1.9) | 0.01 |
| Albumin < 2.5, No. (%) | 3437 (8) | 4402 (9.2) | <0.001 |
| LDH/albumin ratio, median (IQR) | 127.5 (101.1-168.5) | 119.4 (93.7-158.9) | <0.001 |
| LDH/albumin > 150, No. (%) | 13241 (34) | 12528 (29.3) | <0.001 |
|  | | | |

| **Table 6:** LDH | | | |
| --- | --- | --- | --- |
|  | **Infection from pulmonary source**  n=44,491 | **Infection from other sources**  n=50,486 | **P-value** |
| Count, median (IQR) | 2 (1-2) | 2 (1-3) | <0.001 |
| First (U/L), median (IQR) | 418 (344-522) | 385 (316-483) | <0.001 |
| LDH (first) categories, No. (%) |  |  | <0.001 |
| < 480 | 29625 (66.6) | 37557 (74.4) |  |
| 480 – 700 | 11425 (25.7) | 9910 (19.6) |  |
| 700 - 900 | 2279 (5.1) | 1896 (3.8) |  |
| > 900 | 1162 (2.6) | 1123 (2.2) |  |
| 7 days after diagnosis | | | |
