# Supplementary material for: The role of lactate dehydrogenase in hospitalized patients, comparing those with pulmonary versus non-pulmonary infections: A nationwide study
Source: PLoS One. 2023 Mar 29;18(3):e0283380. doi: 10.1371/journal.pone.0283380 (PMC10058092; doi:10.1371/journal.pone.0283380)
Supplement: Attachment — Submitted filename: Response to Reviewers.docx [file pone.0283380.s002.docx]

***Reviewer 1 report:***

*We thank the reviewer for the thorough and incisive review of our manuscript. Our responses to the comments are listed below.*

***Comment # 1***

a well conducted study with a good number of studied patients and very clearly written paper. I think the practical application will be of limited impact on the management of *the* hospitalized patients regarding both diagnosis and treatment

***Our response:***

We concur with the reviewer's perspective that the article is not intended to alter diagnosis or treatment, but rather to serve as a convenient bedside resource for prognostic purposes.

***Reviewer 2 report:***

*We appreciate the reviewer’s positive evaluation of our manuscript and the important comment, to which we responded below*

Thank you for the opportunity to review this interesting study. Authors have performed retrospective review of inpatients admitted with infection between 2001-2020 to understand relationship of lactate dehydrogenase in regarding to outcomes in pulmonary and non-pulmonary infections. I have few suggestions listed below to improve the manuscript:

***Comment # 1***

In this study, authors have shown pulmonary and nonpulmonary infections in an unadjusted model (table 1) only. Also, baseline characteristics (table 1) suggests that both the populations are inherently non-comparable without adjusted analysis. In the discussion 'We also found that the median serum LDH level among patients with pulmonary infections was higher than that of patients with non-pulmonary infections (418 U/L vs. 385 U/L, p<0.001).' may not be correctly interpretable. Author should emphasize this limitation.

***Our response:***

We agree with the reviewer that this issue should be emphasized, and edited the discussion section accordingly:

"The main finding of our study is that elevated LDH levels in hospitalized patients with infectious diseases were associated with a higher mortality rate and more target organ damage, regardless of whether the source of infection was pulmonary or non-pulmonary. Additionally, our univariate non-adjusted analysis revealed a higher median LDH level in patients with pulmonary infections (418 U/L) compared to those with non-pulmonary infections (385 U/L), with a p-value of <0.001."

***Comment # 2***

In table 2, please explain, if gender was added in the model ?

***Our response***:

We acknowledge the reviewer's suggestion that gender could have potentially influenced the presentation of our model. However, our analysis did not identify gender as a statistically significant factor, and thus it was not incorporated into the model presented in Table 2.

***Comment # 3***

I would encourage authors to add univariate analysis in the supplementary tables for transparency of selection of variables in the multivariate analysis.

***Our response***:

We agree with the reviewer and added the following tables (univariate analysis) as supplementary tables:

| **Table 2: Target organ damage** | | | |
| --- | --- | --- | --- |
|  | **Infection from pulmonary source**  **n=44,491** | **Infection from other sources**  **n=50,486** | **P-value** |
| **Respiratory failure, No. (%)** | **2341 (5.3)** | **522 (1)** | **<0.001** |
| **Vascular, No. (%)** | **303 (0.7)** | **313 (0.6)** | **0.24** |
| **Renal, No. (%)** | **3074 (6.9)** | **4194 (8.3)** | **<0.001** |
| **Liver, No. (%)** | **59 (0.1)** | **336 (0.7)** | **<0.001** |
| **Hematologic, No. (%)** | **617 (1.4)** | **631 (1.2)** | **0.06** |
| **Metabolic, No. (%)** | **358 (0.8)** | **71 (0.1)** | **<0.001** |
| **CNS, No. (%)** | **24 (0.1)** | **79 (0.2)** | **<0.001** |
| **SIRS, No. (%)** | **322 (0.7)** | **252 (0.5)** | **<0.001** |
| **Any target organ damage, No. (%)** | **6230 (14)** | **5810 (11.5)** | **<0.001** |
|  | | | |

| **Table 1:** Demographic characteristics and hospitalization details | | | |
| --- | --- | --- | --- |
|  | **Infection from pulmonary source**  n=44,491 | **Infection from other sources**  n=50,486 | **P-value** |
| Age, mean ± SD | 70.7 ± 17.3 | 68 ± 19.4 | <0.001 |
| Male sex, No. (%) | 23226 (52.2) | 23568 (46.7) | <0.001 |
| Deceased, No. (%) | 26651 (59.9) | 26031 (51.6) | <0.001 |
| Age, mean ± SD | 81.1 ± 11.9 | 81.8 ± 11.4 | <0.001 |
| In hosp, No. (%) | 1846 (6.9) | 890 (3.4) | <0.001 |
| One week disch, No. (%) | 478 (1.8) | 427 (1.6) | 0.18 |
| One month disch, No. (%) | 1782 (6.7) | 1779 (6.8) | 0.5 |
| ICU transfer, No. (%) | 934 (2.1) | 547 (1.1) | <0.001 |
| Hospitalization length of stay, median (IQR) | 5 (3-7) | 5 (3-7) | <0.001 |
|  |  |  |  |
|  | | | |

| **Table 3:** Background medical conditions | | | |
| --- | --- | --- | --- |
|  | **Infection from pulmonary source**  n=44,491 | **Infection from other sources**  n=50,486 | **P-value** |
| DM | 16147 (36.3) | 18821 (37.3) | 0.002 |
| IHD | 16070 (36.1) | 15516 (30.7) | <0.001 |
| CVA | 2348 (5.3) | 2954 (5.9) | <0.001 |
| Hematologic malignancy | 2350 (5.3) | 1812 (3.6) | <0.001 |
| Solid malignancy | 24835 (55.8) | 27775 (55) | 0.01 |
| Liver cirrhosis | 534 (1.2) | 919 (1.8) | <0.001 |
| CHF | 10899 (24.5) | 8434 (16.7) | <0.001 |
| CKD/renal failure | 10151 (22.8) | 11435 (22.6) | 0.54 |
| COPD | 15304 (34.4) | 6200 (12.3) | <0.001 |
| HTN | 28915 (65) | 31893 (63.2) | <0.001 |
| Asthma | 8111 (18.2) | 4649 (9.2) | <0.001 |
| A. Fib/Flutter | 9596 (21.6) | 8114 (16.1) | <0.001 |
|  | | | |

| **Table 4:** Chronic medication use | | | |
| --- | --- | --- | --- |
|  | **Infection from pulmonary source**  n=44,491 | **Infection from other sources**  n=50,486 | **P-value** |
| BB | 15156 (34.1) | 16127 (31.9) | <0.001 |
| ACEi | 13103 (29.5) | 14917 (29.5) | 0.75 |
| ARB’s | 5246 (11.8) | 5161 (10.2) | <0.001 |
| Steroids | 7367 (16.6) | 4648 (9.2) | <0.001 |
| Statins | 16674 (37.5) | 17828 (35.3) | <0.001 |
| Altroxin | 2775 (6.2) | 2951 (5.8) | 0.01 |
| Purchased three months before hospitalization | | | |

| **Table 5:** Lab tests results during hospitalization (limiting values) | | | |
| --- | --- | --- | --- |
|  | **Infection from pulmonary source**  n=44,491 | **Infection from other sources**  n=50,486 | **P-value** |
| Hb (min), gr/dL, mean ± SD | 11.4 ± 1.9 | 11.2 ± 1.9 | <0.001 |
| Hb < 6, No. (%) | 78 (0.2) | 95 (0.2) | 0.67 |
| Hb < 7, No. (%) | 495 (1.1) | 569 (1.1) | 0.9 |
| Hb < 9, No. (%) | 4888 (11.1) | 5997 (12) | <0.001 |
| WBC (max), 10^3^/µL, median (IQR) | 11.9 (8.8-16.2) | 12.6 (9.2-16.8) | <0.001 |
| WBC (min), 10^3^/µL, median (IQR) | 8.1 (6.1-10.5) | 7.9 (6-10.3) | <0.001 |
| WBC > 15, No. (%) | 13476 (30.6) | 17065 (34) | <0.001 |
| WBC < 5, No. (%) | 6019 (13.9) | 6670 (13.4) | 0.06 |
| PLT (min), 10^3^/µL, mean ± SD | 219.2 ± 87.5 | 212.5 ± 83.2 | <0.001 |
| PLT < 50, No. (%) | 742 (1.7) | 622 (1.3) | <0.001 |
| INR (max), median (IQR) | 1.1 (1-1.3) | 1.1 (1-1.3) | <0.001 |
| Glucose (max), mg/dL, median (IQR) | 147 (120-195) | 140 (115-186) | <0.001 |
| Glucose (min), mg/dL, median (IQR) | 97 (84-117) | 94 (82-112.7) | <0.001 |
| Glucose > 200, No. (%) | 10175 (23.3) | 10186 (20.6) | <0.001 |
| Glucose < 50, No. (%) | 545 (1.3) | 719 (1.5) | 0.008 |
| Glucose < 70, No. (%) | 3105 (7.1) | 4463 (9) | <0.001 |
| Glucose coefficient of variance, median (IQR) | 0.2 (0.1-0.3) | 0.2 (0.1-0.3) | 0.008 |
| AST (max), U/L, median (IQR) | 26 (19-41) | 25 (18-41) | <0.001 |
| ALT (max), U/L, median (IQR) | 22 (15-38) | 21 (14-36) | <0.001 |
| Creatinine (max), mg/dL, median (IQR) | 1 (0.8-1.4) | 1 (0.8-1.4) | <0.001 |
| Maximal CRE/first CRE ratio, median (IQR) | 1 (1-1.1) | 1 (1-1) | <0.001 |
| Maximal CRE/first CRE > 2, No. (%) | 515 (1.2) | 451 (0.9) | <0.001 |
| D-Dimer (max), ng/mL, median (IQR) | 656 (5.6-1509.9) | 450.5 (1.7-1576) | 0.002 |
| Urea (max), mg/dL, median (IQR) | 47 (33-72) | 44 (30.5-67) | <0.001 |
| Albumin (min), gr/dL, median (IQR) | 3.3 (2.9-3.7) | 3.3 (2.9-3.6) | <0.001 |
| Albumin < 1.5, No. (%) | 43 (0.1) | 46 (0.1) | 0.86 |
| Albumin < 2, No. (%) | 720 (1.7) | 909 (1.9) | 0.01 |
| Albumin < 2.5, No. (%) | 3437 (8) | 4402 (9.2) | <0.001 |
| LDH/albumin ratio, median (IQR) | 127.5 (101.1-168.5) | 119.4 (93.7-158.9) | <0.001 |
| LDH/albumin > 150, No. (%) | 13241 (34) | 12528 (29.3) | <0.001 |
|  | | | |

| **Table 6:** LDH | | | |
| --- | --- | --- | --- |
|  | **Infection from pulmonary source**  n=44,491 | **Infection from other sources**  n=50,486 | **P-value** |
| Count, median (IQR) | 2 (1-2) | 2 (1-3) | <0.001 |
| First (U/L), median (IQR) | 418 (344-522) | 385 (316-483) | <0.001 |
| LDH (first) categories, No. (%) |  |  | <0.001 |
| < 480 | 29625 (66.6) | 37557 (74.4) |  |
| 480 – 700 | 11425 (25.7) | 9910 (19.6) |  |
| 700 - 900 | 2279 (5.1) | 1896 (3.8) |  |
| > 900 | 1162 (2.6) | 1123 (2.2) |  |
| 7 days after diagnosis | | | |

***Comment # 4***

In the table 2, 3, and 4, authors have added many biomarkers such as Hb, WBC, PLT, and Albumin. And all these biomarkers have significant associations. However, authors have not discussed the potential role/importance of these markers. I would suggest adding more discussion about these biomarkers.

***Our response***:

We agree with the reviewer and edited the discussion section:

"…. Although leukopenia is widely recognized in the literature as a marker for mortality in patients with infection(1), our study yielded unexpected results, as leukopenia was not found to be associated with mortality. Instead, it was linked to increased target organ damage. In contrast, anemia and thrombocytopenia, which have been consistently associated with adverse outcomes in previous studies (2), were found to be correlated with both mortality and target organ damage. Similarly, low levels of albumin were significantly associated with all outcomes, as previously demonstrated in a recently published research (3).

1. Samuel H. Belok, Nicholas A. Bosch, Elizabeth S. Klings, Allan J. Walkey. Evaluation of leukopenia during sepsis as a marker of sepsis-defining organ dysfunction. PLoS One. 2021; 16(6): e0252206. doi: 0.1371.0252206.

2. Mehdi Mirsaeidi, Paula Peyrani, Stefano Aliberti, Giovanni Filardo PhD d et al. Thrombocytopenia and Thrombocytosis at Time of Hospitalization Predict Mortality in Patients With Community-Acquired Pneumonia. Chest Original Research Volume 137, Issue 2, February 2010, Pages 416-420

3. Frenkel A, Novack V, Bichovsky Y, Klein M, Dreiher J. Serum Albumin Levels as a Predictor of Mortality in Patients with Sepsis: A Multicenter Study. Isr Med Assoc J, 2022 Jul ;24:454-459

***Comment # 5***

In the conclusion section, 'Additionally, patients with pulmonary infections and LDH levels >900 U/L are at particularly increased risk of death within 30 days. The findings presented here suggest LDH testing as a helpful bedside tool to estimate mortality risk in patients with infectious disease.' I would suggest rephrasing these sentences, as this study did not assess risk but 'increase odds of death'.

***Our response***:

We concur with the reviewer's observation that the original phrasing of the paragraph was imprecise and potentially misleading. Therefore, we have revised the language as follows:

"…. Additionally, the analysis revealed that patients with pulmonary infections and LDH levels greater than 900 U/L had higher mortality odds within a 30-day timeframe. These findings support the utility of LDH testing as a valuable bedside tool for estimating mortality odds in patients with infectious diseases."

***Comment # 6***

A number of previous studies and reviews have shown that In patients with COVID-19, the increased LDH level is associated with a higher risk of negative clinical prognosis and higher mortality. Current data included the year 2020 in the analysis. Author should add a caveat specific diseases such as COVID-19.

***Our response***:

We agree with the reviewer, and edited the discussion section accordingly:

"…It is worth noting that the current study did not involve patients with COVID-19 infection, despite data being collected up until 2020. However, it is noteworthy that recent research has highlighted a significant association between high LDH values and disease severity and mortality among COVID-19 patients. [18]."

[18]. Brandon Michael Henry , Gaurav Aggarwal , Johnny Wong , Stefanie Benoit et al. Lactate dehydrogenase levels predict coronavirus disease 2019 (COVID-19) severity and mortality: A pooled analysis. Am J Emerg Med. 2020 Sep;38(9):1722-1726. doi: 10.1016/j.ajem.2020.05.073. Epub 2020 May 27.

***Comment # 7***

I will suggest to include more references to support the statements made in the discussion. I will also suggest to review the manuscript for grammar and use same fonts for manuscript and all references.

***Our response***:

We appreciate the reviewer's contribution in reviewing the manuscript for grammatical errors and ensuring consistency in fonts. Furthermore, we have incorporated four additional references to support the arguments presented in the discussion, while all grammatical inaccuracies identified during the review process have been rectified.

Samuel H. Belok, Nicholas A. Bosch, Elizabeth S. Klings, Allan J. Walkey. Evaluation of leukopenia during sepsis as a marker of sepsis-defining organ dysfunction. PLoS One. 2021; 16(6): e0252206. doi: 0.1371.0252206.

1) Mehdi Mirsaeidi, Paula Peyrani, Stefano Aliberti, Giovanni Filardo PhD d et al. Thrombocytopenia and Thrombocytosis at Time of Hospitalization Predict Mortality in Patients With Community-Acquired Pneumonia. Chest Original Research Volume 137, Issue 2, February 2010, Pages 416-420
2) Frenkel A, Novack V, Bichovsky Y, Klein M, Dreiher J. Serum Albumin Levels as a Predictor of Mortality in Patients with Sepsis: A Multicenter Study. Isr Med Assoc J, 2022 Jul ;24:454-459
3) Brandon Michael Henry , Gaurav Aggarwal , Johnny Wong , Stefanie Benoit et al. Lactate dehydrogenase levels predict coronavirus disease 2019 (COVID-19) severity and mortality: A pooled analysis. Am J Emerg Med. 2020 Sep;38(9):1722-1726. doi: 10.1016/j.ajem.2020.05.073. Epub 2020 May 27.

***Reviewer 3 report:***

Thorough, intelligible review of elevated LD in sepsis with meaningful, interesting results.
Possibly limited clinical utility given the increased LD in different kind of sepsis only modest and would add little to clinical evaluation and management.
There is a considerable literature in this area, which they only summarise but usefully compare to other biomarkers.

***Our response***:

We agree with the reviewer. Following his comment, we expanded the discussion while referring to other biomarkers. We also added four references following this expansion.

1..Samuel H. Belok, Nicholas A. Bosch, Elizabeth S. Klings, Allan J. Walkey. Evaluation of leukopenia during sepsis as a marker of sepsis-defining organ dysfunction. PLoS One. 2021; 16(6): e0252206. doi: 0.1371.0252206.

2. Mehdi Mirsaeidi, Paula Peyrani, Stefano Aliberti, Giovanni Filardo PhD d et al. Thrombocytopenia and Thrombocytosis at Time of Hospitalization Predict Mortality in Patients With Community-Acquired Pneumonia. Chest Original Research Volume 137, Issue 2, February 2010, Pages 416-420

3. Frenkel A, Novack V, Bichovsky Y, Klein M, Dreiher J. Serum Albumin Levels as a Predictor of Mortality in Patients with Sepsis: A Multicenter Study. Isr Med Assoc J, 2022 Jul ;24:454-459

4. Brandon Michael Henry , Gaurav Aggarwal , Johnny Wong , Stefanie Benoit et al. Lactate dehydrogenase levels predict coronavirus disease 2019 (COVID-19) severity and mortality: A pooled analysis. Am J Emerg Med. 2020 Sep;38(9):1722-1726. doi: 10.1016/j.ajem.2020.05.073. Epub 2020 May 27.
